# The Essence of Palliative Care Is Best Viewed as the “Problematization”

**DOI:** 10.1089/jpm.2018.0501

**Published:** 2019-01-28

**Authors:** Atsushi Mizuno, Tatsuhiro Shibata, Shogo Oishi

**Affiliations:** ^1^ Department of Cardiology, St.Luke's International Hospital, Tokyo, Japan.; ^2^ Division of Cardiovascular Medicine, Department of Internal Medicine, Kurume University School of Medicine, Tokyo, Japan.; ^3^ Department of Cardiology, Himeji Cardiovascular Center, Himeji, Japan.

Dear Editor:

Palliative care was first defined by World Health Organization (WHO) in 1989, as care especially for “patients whose disease is not responsive to curative treatment.”^1^ WHO further broadened the target population to “the patients and their families facing the problems associated with life-threatening illness” in 2002.^2^ Furthermore, the scope of palliative care has become even broader over time, which resulted in the idea that “palliative care” should be delivered regardless of the diagnosis and prognosis, the time, and the place/situation.^3^ Although cardiologists totally agree that the key concept of palliative care by WHO should be improving the patients' quality of life (QOL), we have difficulty differentiating between “palliative care” and “medicine” itself, which are overlapped significantly. We believe that this is the reason why Cairns W stated that “no other area of health care seems to have gone to such lengths to write definitions of itself as has palliative care.”^4^

In spite of the recent attention to palliative care for noncancer patients, such as those who suffer from heart disease, the definition of palliative care is too comprehensive and vague for many nonpalliative care specialists, such as cardiologists and primary care physicians, to understand. We better comprehend the essence of palliative care as a “problematization” to improve the patients' QOL, which is consistent with current professional knowledge and availability. Problematization in medicine refers to the question of “what is a current problem that I as health care provider can do to improve patients' QOL?”^5^ Admitting that problematization is the essence of palliative care, the historically broadening scope can be explained and easier to understand.

Several decades ago, we initially developed “palliative care” to cope with not only the physical pain that patients experienced but also other pains, such as spiritual ones, to resolve problems that existed. There was a gap between the support that medicine provided in that era and the patients' needs. For example, early-stage cancer patients faced pains that medicine was unprepared to care for appropriately. Now, considering the plateau diagnostic capacity and treatment efficacy for noncancer patients with chronic diseases, such as heart failure and respiratory disease, new issues have developed related to clinical decision making under uncertain outcomes. Therefore, the chief role of palliative care now might be “advance care planning” and support in the decision-making process in addition to pain relief. In this way, the scope of palliative care has changed as a result of the external situation and resource availability. Therefore, the concept of “problematization” as the essence of palliative care might make it easier for clinicians to understand rather than basing their understanding on the WHO definition of palliative care.

## References

[1] World Health Organization: Cancer Pain Relief and Palliative Care: Report of a WHO Expert Committee [Meeting Held in Geneva from 3 to 10 July 1989]. World Health Organization, Geneva, Switzerland, 1990 1702248

[2] Pastrana T, Jünger S, Ostgathe C, et al.: A matter of definition–key elements identified in a discourse analysis of definitions of palliative care. Palliat Med 2008;22:222–232 18477716 10.1177/0269216308089803

[3] Worldwide Palliative Care Alliance, World Health Organization: Global Atlas of Palliative Care at the End of Life. London: Worldwide Palliative Care Alliance, 2014

[4] Cairns W: The problem of definitions. Prog Palliat Care 2001;9:187–189

[5] Harfield S: On design ‘problematization’: Theorising differences in designed outcomes. Des Stud 2007;28:159–173

